# 

**Published:** 1860-05

**Authors:** 


### CONTENTS FOR MAY, 1860.

### ORIGINAL COMMUNICATIONS.

Croup and Diphtheria. By ORREN SMITH, M. D., of Chicago,.......... 257
Benign Cartilaginous Tumors. By E. ANDREWS, M. D., of Chicago,.......... 270
Correspondence on Typhoid Fever. By J. A. BRENNEMAN. M. D., Ill.......... 271
Concentrated Organic Medicines. By V. L. HURLBUT, of Chicago,.......... 276
A Case of Poisoning and Recovery from the Use of Veratrum Viride. By N. O. PEARSON, M. D., Palestine, Ill.......... 277
Cliniques. By Prof. N. S. DAVIS,.......... 278—283
Proceedings Chicago Academy Medical Sciences,.......... 287

### BOOK AND PAMPHLET NOTICES.

Gross' System of Surgery,.......... 291
A Practical Treatise on Fractures and Dislocations.......... 292
A Medico-Legal Treatise on Malpractice and Medical Evidence.......... 293
The Diseases of the Ear.......... 295
The London Lancet,.......... 296
American Journal of Medical Sciences.......... 296

### SELECTIONS.

The Auricles of the Heart act by their Elasticity and Contractility. not by Muscles,.......... 297
New Disinfectant for Dressing of Gangrenous Wounds, Ulcers, etc.,.......... 298
Preservation of Bodies for Scientific purposes,.......... 300
Uterine Polypsus treated by injections of the Perchloride of Iron,.......... 300
Florence Nightingale and her "Notes on Nursing,.......... 301
Sanitary Science,.......... 302
Advantages of the Use of Glycerine in Surgery,.......... 303
Iodized Glycerine in Skin Diseases,.......... 304
Chloroform in Reduction of Dislocations,.......... 304
Perchloride of Iron in Diphtheritis,.......... 305
Arsenious Acid in Apoplectic Congestions,.......... 306
Treatment of Prolapsus of the Funus,.......... 307
Gelsemin.......... 307
Hypophosphites in Phthisis,.......... 308
Gastrotomy,.......... 308
Iodide of Potassium in Diseases of Brain in Children,.......... 308
Puerperal Convulsions.......... 309
Podophyllin and Leptandrin as a substitute for Mercurials in diseases of the digestive Organs,.......... 310
Veratrum Viride in cases of Children,.......... 312

### EDITORIAL.

Chicago Medical Journal and State Medical Society.......... 314
Chicago Medical Journal and Dr. E. B. Wolcott,.......... 317
American Medical Association,.......... 318
Tobacco, Coffee and Tea,.......... 318

